# Bioelectronic Delivery of Potassium Ions Controls Membrane Voltage and Growth Dynamics in Bacteria Biofilms

**DOI:** 10.1007/s44174-024-00209-w

**Published:** 2024-07-02

**Authors:** Harika Dechiraju, Yixiang Li, Colin Comerci, Le Luo, Sydnie Figuerres, Niloofar Asefi, Ansel Trevino, Alexie Barbee, Maryam Tebyani, Prabhat Baniya, Mircea Teodorescu, Gürol Süel, Marco Rolandi

**Affiliations:** 1 Department of Electrical and Computer Engineering, University of California Santa Cruz, Santa Cruz, CA 95060, USA; 2 Division of Biological Sciences, University of California San Diego, La Jolla, CA 92093, USA; 3 UC Santa Cruz Genomics Institute, University of California Santa Cruz, Santa Cruz, CA 95060, USA; 4 Institute for the Biology of Stem Cells, University of California Santa Cruz, Santa Cruz, CA 95064, USA

**Keywords:** Bioelectronics, Membrane potential, Biofilms

## Abstract

Bioelectrical signaling, or bioelectricity, is crucial in regulating cellular behavior in biological systems. This signaling, involving ion fluxes and changes in membrane potential (V_mem_), is particularly important in the growth of bacterial biofilm. Current microfluidic-based methods for studying bacterial colonies are limited in achieving spatiotemporal control over ionic fluxes due to constant flow within the system. To address this limitation, we have developed a platform that integrates biofilm colonies with bioelectronic ion pumps that enable delivery of potassium (K^+^) ions, allowing for controlled manipulation of local potassium concentration. Our study examines the impact of controlled K^+^ delivery on bacterial biofilm growth patterns and dynamics. We observed significant changes in V_mem_ and coordination within the biofilms. Furthermore, we show that localized K + delivery is highly effective in controlling biofilm expansion in a spatially targeted manner. These findings offer insights into the mechanisms underlying bacterial signaling and growth, and suggest potential applications in bioengineering, synthetic biology, and regenerative medicine, where precise control over cellular signaling and subsequent tissue growth is required.

## Introduction

Endogenous electrical signaling within cells, bioelectricity, is crucial for understanding and regulating cell behavior [1, 2]. An example is the transmission of action potentials in neurons [3]. Bioelectrical signaling is not limited to electrically excitable cells and is generally involved in the modulation of cell function, metabolism, and morphogenesis, a feature that has been exploited in bioengineering, regenerative medicine, and synthetic morphology applications [4, 5]. Recent research indicates that bioelectrical signaling is widespread across various biological organisms including bacteria [6]. Bacterial biofilms, consisting of densely packed cells, exhibit metabolic coordination facilitated by electrochemical signaling [7]. Within these biofilms, metabolic changes lead to alterations in individual cells of the membrane potential (V_mem_), which represents the electrochemical potential difference across the cell membrane [7]. Changes in V_mem_ involve the movement of charged species across the cell membrane, in particular cations such potassium (K^+^) [7]. Numerous studies have highlighted the role of potassium in modulating the membrane potential of *Bacillus subtilis,* influencing the formation of biofilms, as well as mediating long-range electrical signaling in bacterial communities [7-9]. Current methods for studying and perturbing bacterial colonies predominantly rely on microfluidic systems [10-12]. One limitation of these systems is achieving precise spatiotemporal control over ionic fluxes and small molecule concentrations. This remains a challenge in microfluidic platforms due to the presence of constant flow involved in delivering fluid that contains the ions of interest.

**Fig. 1:**
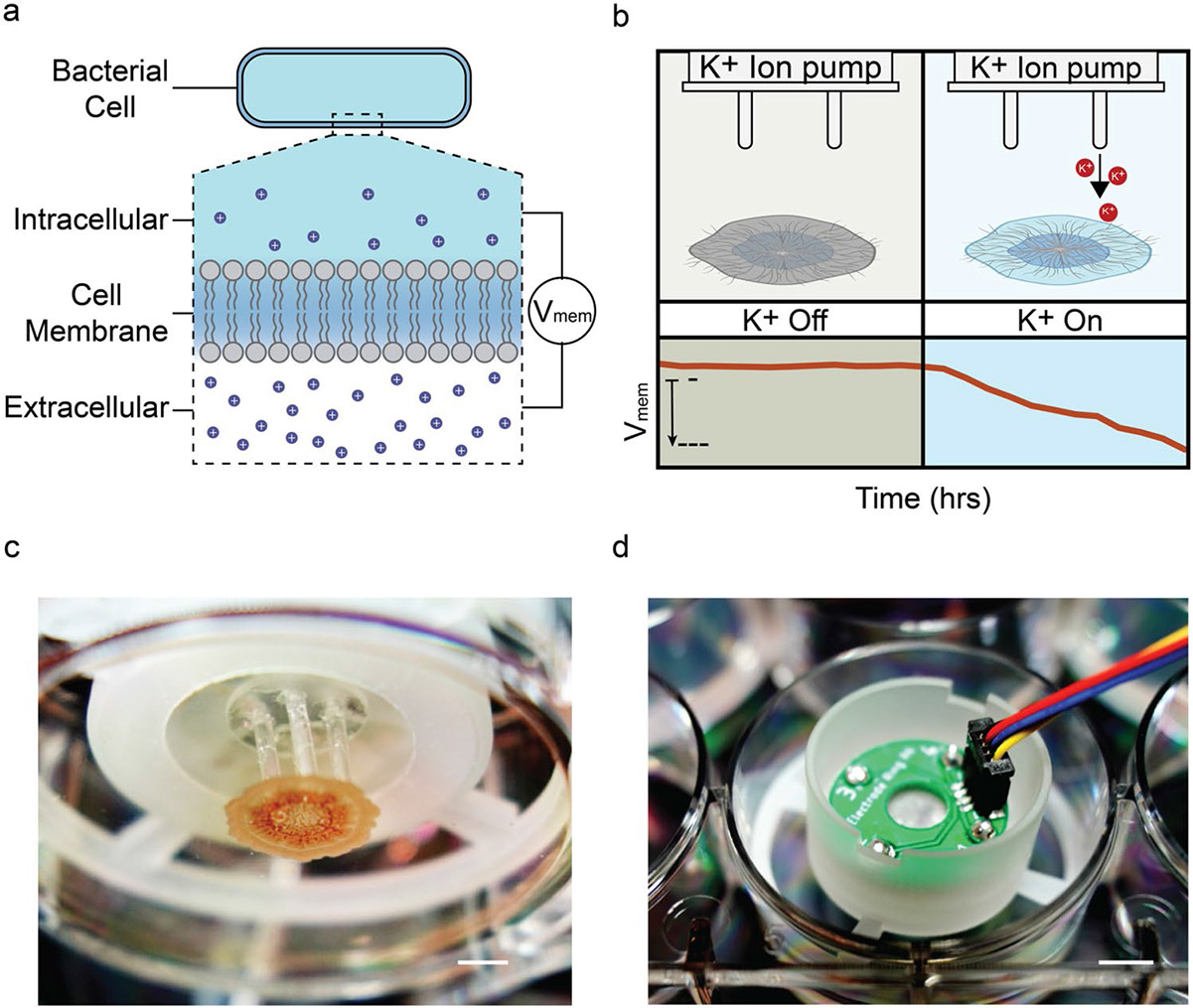
**a** Schematic of bacterial cell and membrane potential arising from an ion concentration gradient across the cell membrane (V_mem_). **b** V_mem_ response of a bacteria biofilm to external K^+^ delivered from an ion pump. **c** View from the bottom of a 6-well plate, highlighting the integration of an ion pump with biofilm. The capillaries of the ion pump are in contact with the biofilm. The biofilm occupies the orange region within the image. Scale bar = 3 mm. **d** Image of the integrated ion pump from a top-down perspective. The device is designed to directly sit in a 6-well plate through the adapter. Scale bar = 5 mm

Bioelectronics merges materials and devices for bridging signaling in biological systems, ions and biochemicals, with electronics [13-16]. To this end, in bioelectronic ion pumps an electronic signal deliver ions and biochemicals from a reservoir to modulate the function of a target biological system [17-22]. Ion pumps have successfully delivered ions such as H^+^, K^+^ and biochemicals such as GABA, Fluoxetine to 2D and 3D cell cultures in vitro and in vivo [20, 23-27]. In this work, we have integrated colonies of *Bacillus subtilis* with bioelectronic ion pumps able to deterministically deliver K^+^ ions locally to the bacterial biofilm. With this system, we studied how local and timed K^+^ delivery regulates bacteria bioelectrical communication via V_mem_ and how this communication modifies their growth patterns and dynamics (Fig. 1).

## Results and Discussion

The membrane voltage (V_mem_) in *B. subtilis* is the potential difference across the cell membrane (Fig. 1a). V_mem_ arises from differences in the electrochemical gradient of ions as well as ion fluxes across the membrane. For *B. subtilis* the resting V_mem_ is approximately −150 mV and K^+^ concentration differences across the membrane play a major role in V_mem_ regulation [7, 28, 29]. Extracellular changes and oscillations in [K^+^] result in changes and oscillations in V_mem_ and vice versa [K^+^] [7, 28, 29]. Here, we wanted to look into the dynamics of the relationship between [K^+^] and V_mem_ in *B. subtilis* biofilms aided by bioelectronic delivery of K^+^ with spatiotemporal control (Fig. 1b). To this end, we grew a biofilm-forming strain of B. subtilis on an agar substrate in a six-well plate that incorporated an engineered ion pump designed to deliver K^+^ [23]. This ion pump delivers K^+^ through hydrogel filled glass capillaries that are positioned directly above the *B. subtilis* biofilm (Fig. 1c). The pump and capillaries are held in position with a custom-made holder that fixes the ion pump and the on-board electronics to the sides of the six well plate [23] (Fig. 1d).

**Fig. 2:**
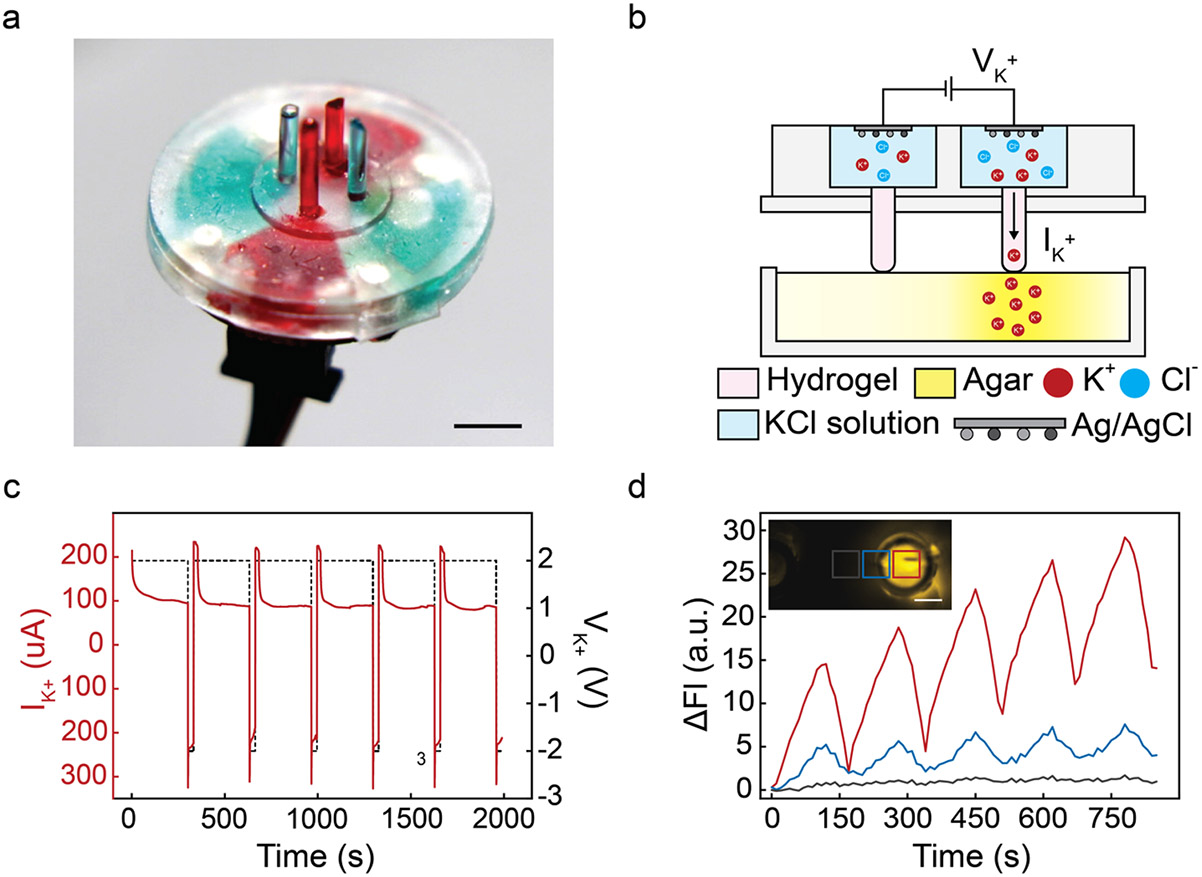
**a** Ion pump with the reservoirs and capillaries highlighted using food color. The reservoirs and capillaries in red correspond to the WE of the ion pump and the reservoirs and capillaries in blue correspond to the CE. Scale bar = 4 mm. **b** Schematic of the potassium ion pump mechanism, illustrating the direction of potassium ion movement during the actuation. Agar substrate is infused with the yellow-green IPG-4 fluorescent dye, where the brightness of yellow indicates the intensity of fluorescence. The fluorescence response is utilized to report the [K +]. **c** Current response of the ion pump for a series of alternating V_K+_. **d** Spatial resolution of K^+^ delivery by the ion pump. The fluorescent intensities of the different locations in the proximity of the tip of Working Electrode (WE) over 5 actuation cycles are given. The inset indicates the location of interest, which are inside the tip (red), on the edge of the tip (blue), outside the tip (gray) respectively. Scale bar = 400 μm

The bioelectronic ion pump comprises four reservoirs made of PDMS filled with an aqueous solution of 1 M KCl (Fig. 2a). Each reservoir is linked to the target solution via an 800 μm diameter glass capillary filled with a cation-exchange hydrogel of 2-Acrylamido-2-methylpropane sulfonic acid: Poly (ethylene glycol) diacrylate (AMPSA: PEGDA) (Fig. 2a, b). Within the reservoirs, Ag and AgCl wires serve as the working (WE) and reference/counter electrodes (RE/CE) respectively. V_K+_ is the potential difference measured between WE and RE/CE. A positive V_K+_ drives K^+^ ions from the reservoir into the target solution containing the biofilm, as previously described. [23] An anionic hydrogel, serving as ion exchange membrane, ensures that only K^+^ and not Cl^−^ is transferred to and from the target. [23] To test the ion pump, we first used a series of alternating V_K+_ pulses of +2 V for 300 s and −2 V for 30 s to evaluate the delivery efficiency. At a V_K+_ = 2 V, we recorded an ionic current I_K+_ = 100μA (Fig. 2c) indicating that K^+^ are transferring from the reservoir to the target solution. We measured K^+^ delivery from the ion pump with the fluorescence dye IPG-4, an extracellular K^+^ indicator (Supplementary Fig. S3) [30, 31]. The fluorescence intensity of IPG-4 increased with [K^+^] [30, 31]. With appropriate calibration (Supplementary Figs. S3 and S4), measuring the fluorescence intensity of IPG-4 yields a quantitative measurement of Δ[K^+^] in the target solution. Five V _K+_ pulses (V_K+_ = 2 V, t = 120 s; V_K+_ = −2 V, t = 50 s) increased [K^+^] in the target solution (Fig. 2d). We expect an increase in [K^+^] because the duty cycle for V_K+_ = 2 V, which delivers K^+^ to the target solution, is longer than for V_K+_ =−2 V, which removes K^+^ from the solution. At the end of this sequence, Δ[K^+^] = 45 mM as reflected by an overall increase in IPG-4 fluorescence (Fig. 2d). The use of V _K+_ = −2 V as part the delivery sequence is counterintuitive at first because, during V _K+_ =−2 V, K.^+^ is removed from the target solution as indicated by a temporary drop in IPG-4 fluorescence (Fig. 2d). However, from our experience, a pulse sequence with both positive and negative V _K+_ results in a higher delivery efficiency. [23] We have not quantified this phenomenon yet, however, capacitive effects at the electrode double layer interface as well as ion exchange inside the hydrogel may be involved [23, 32].

It is worth noting fluorescence intensity near the WE is stronger than the fluorescence intensity further away from the WE (Fig. 2d). This observation indicates that the bioelectronic ion pump controls the delivery of ions with a temporal resolution that is higher than the diffusion timeframe for K^+^ to diffuse in the solution contained in the well plate. The temporal control of the delivery also ensures that the ion pump affects [K^+^] in an area predominantly directly below the capillary that contains the working electrode in an area of (400 μm)^2^ shown as the red square in Fig. 2d. The spatial resolution of the pump can be improved by using smaller capillaries that, however, deliver smaller quantities of K^+^. As we monitor areas further from the WE and closer to the CE (blue and black squares), Δ[K^+^] induced by the ion pumps drops sharply as indicated in the red, blue, and black plots in Fig. 2d. Even 400 μm away from the edge of the capillary (black square), Δ[K^+^] at the end of the cycle is only 4% (2 mM) of Δ[K^+^] = 49 mM directly underneath the outlet of the capillary. Even for the area close to the edge of the capillary (blue square), Δ[K^+^] = 15 mM, which is < 30% of the maximum value of Δ[K^+^]. To confirm our experimental data, we compare the observed [K^+^] as a function of time (Supplementary Fig. S5—top row) with the results from a model created with COMSOL Multiphysics (Supplementary Fig. S5—bottom row). In this model, we employ the outlet of the capillary as a source of K^+^ using the K^+^ delivered from the ion pump. The quantity of K^+^ transported to the agar substrate is calculated by measuring I_K+_ and an estimate of delivery efficiency of ~ 30% as previously described. [23] Once K^+^ reaches the solution, the diffusion of K^+^ in agar is governed by Fick’s laws using a diffusion coefficient for K^+^ in agar of 1.6 × 10^−9^ m^2^ s^−1^ at 20 °C [33]. We then plot the spatial distribution of [K^+^] as a function of time using a color scale that mimics the equivalent response of the IPG-4 dye used in our experiments (Fig. S5 bottom row). From these plots, the spatial distribution of [K^+^] in the experimental data (Fig. 2d) is in qualitative agreement with the spatial distribution in our diffusion model (Fig. S5 bottom row). We enhance this qualitative observation by providing quantitative measurements of [K^+^]. The same square areas (inset of Fig. 2d) are used to calculate the Δ [K^+^] in our model. Δ[K^+^] decreases as a function of distance from WE in the same fashion as the experimental data (Fig. 2d). In our model, Δ[K^+^] = 66 mM, 30 mM, 3 mM in the areas corresponding to the red, blue, and black square in Fig. 2d (Supplementary Fig. S6). The results from the model compare well with Δ[K^+^] = 47 mM, 15 mM, 2 mM for the experimental data. Causes for the small discrepancy include limitations of estimating K^+^ diffusion coefficient, the homogeneity of agar, and the calibration of the [K^+^] fluorescent dye.

**Fig. 3:**
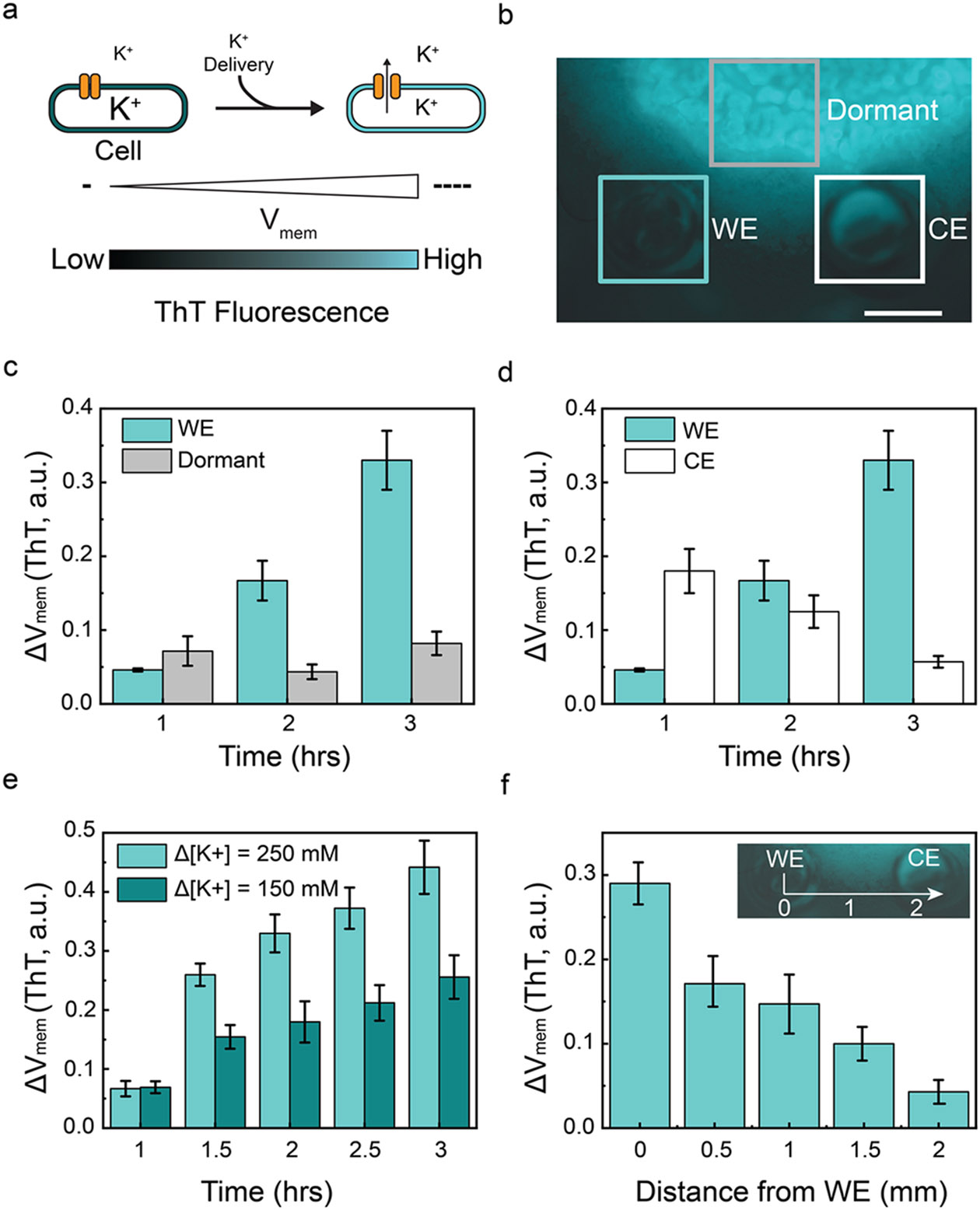
**a** Schematic of the opening of ion channels in the bacterial cell membrane and the correlation between ThT fluorescence and V_mem_. The resting V_mem_ is negative. ThT fluorescence increases when the V_mem_ of the cell become more negative. **b** Biofilm with the Working Electrode (WE), Counter Electrode (CE), and dormant regions highlighted. Scale bar = 800 μm. **c** Temporal evolution of V_mem_ changes observed at the WE and dormant regions. **d** Temporal evolution of V_mem_ changes observed at the WE and CE. **e** Temporal evolution of V_mem_ changes at varying [K^+^] concentrations. **g** Variation in V_mem_ as a function of the distance from the working electrode (WE)

In *Bacillus Subtilis,* under constant conditions, the intracellular [K^+^] of ~ 300 mM is much greater than the media concentration of 8 mM (Fig. 3a) [34, 35]. An increase in extracellular [K^+^] to 150 mM, leads to opening of K^+^ channels, which allows the K^+^ ions to flow from inside the bacteria to the extracellular space. The flow results in a shift of V_mem_ to a more negative value. We measure this change with the Thioflavin-T (ThT) dye as previously described [7]. ThT is a positively charged membrane permeable dye. Thus, ThT can act as a Nernstian potential indicator where a more negative V_mem_ induces more ThT to enter inside the cell, resulting in the increase in fluorescence intensity. To monitor these changes with the ion pump, we integrate the ion pump with a biofilm-forming strain of *Bacillus Subtilis* to investigate the impact of localized changes in extracellular [K^+^] (Fig. 3b). The biofilm comprises two principal segments – a central dormant region (bright in Fig. 3b) and a peripheral active region where bacterial growth occurs. We selected a film in which the position of the Working Electrode (WE) and a Counter/Electrode Reference (CE/RE) puts them in contact with the active biofilm region (Fig. 3b). We then delivered K^+^ from the capillary marked WE using the pulse sequence (V_K+_ = 2.5 V, t = 600 s; V_K+_ = −2.5 V, t = 60 s) for a 2 h period between t = 1 h and t = 3 h.

Initially, we monitor the V_mem_ change of the biofilm at the WE and the dormant region throughout actuation. While the fluorescence intensity of ThT steadily increases at the WE during actuation, the fluorescence intensity of ThT remains largely unchanged at the dormant region. This observation aligns with our expectation that actuation would have no effect on the dormant biofilm region (Fig. 3c). We then compare the impact of actuation at the WE with that at the CE. The V_mem_ of the treated biofilm region increases, evident from the ThT fluorescence intensity increase. This finding corroborates previously demonstrated biofilm behavior [7, 9, 28, 29]. Additionally, an increase in [K^+^] near the WE leads to a slight reduction in [K^+^] at the CE, as the CE reservoir attracts physiological cations due to the corresponding −V_K+_ at that electrode. Consequently, an opposing behavior is observed in the biofilm around the CE (Fig. 3d).

To further assess the effect of [K^+^] on V_mem_, we employ different pulse sequence to the ion pump so as to achieve different K^+^ delivery to the biofilm. Here, V_K+_ = 2.5 V (V_K+_ = 2.5 V, t = 600 s; V_K+_ = −2.5 V, t = 60 s) and V_K+_ = 1.5 V (V_K+_ = 1.5 V, t = 600 s; V_K+_ = −1.5 V, t = 60 s) are used for 2 h, resulting in a final Δ[K^+^] = 250 mM and Δ[K^+^] = 150 mM, respectively. The Δ[K^+^] is estimated by measuring the difference of the fluorescence intensity between the beginning (t = 1 h) and the conclusion (t = 3 h) of the actuation. In Supplementary Fig. S7, we show the change of fluorescence intensity around the WE before and after the actuation, indicating the [K^+^] increases during the actuation. We noted that the ThT intensity rises with increasing [K^+^], with a more pronounced intensity change observed at higher [K^+^]. This suggests that higher [K^+^] induces a greater alteration in the V_mem_ of the biofilm (Fig. 3e).

Finally, to illustrate the impact of the spatial resolution of K^+^ delivery on V_mem_, we plot change in ThT fluorescence intensity (t = 1 h and t = 3 h) hours as a function of the distance from WE along a line that connects the WE to the CE/RE. The intensity of change is highest at the WE and gradually diminishes as we move towards the CE/RE (Fig. 3f).

**Fig. 4:**
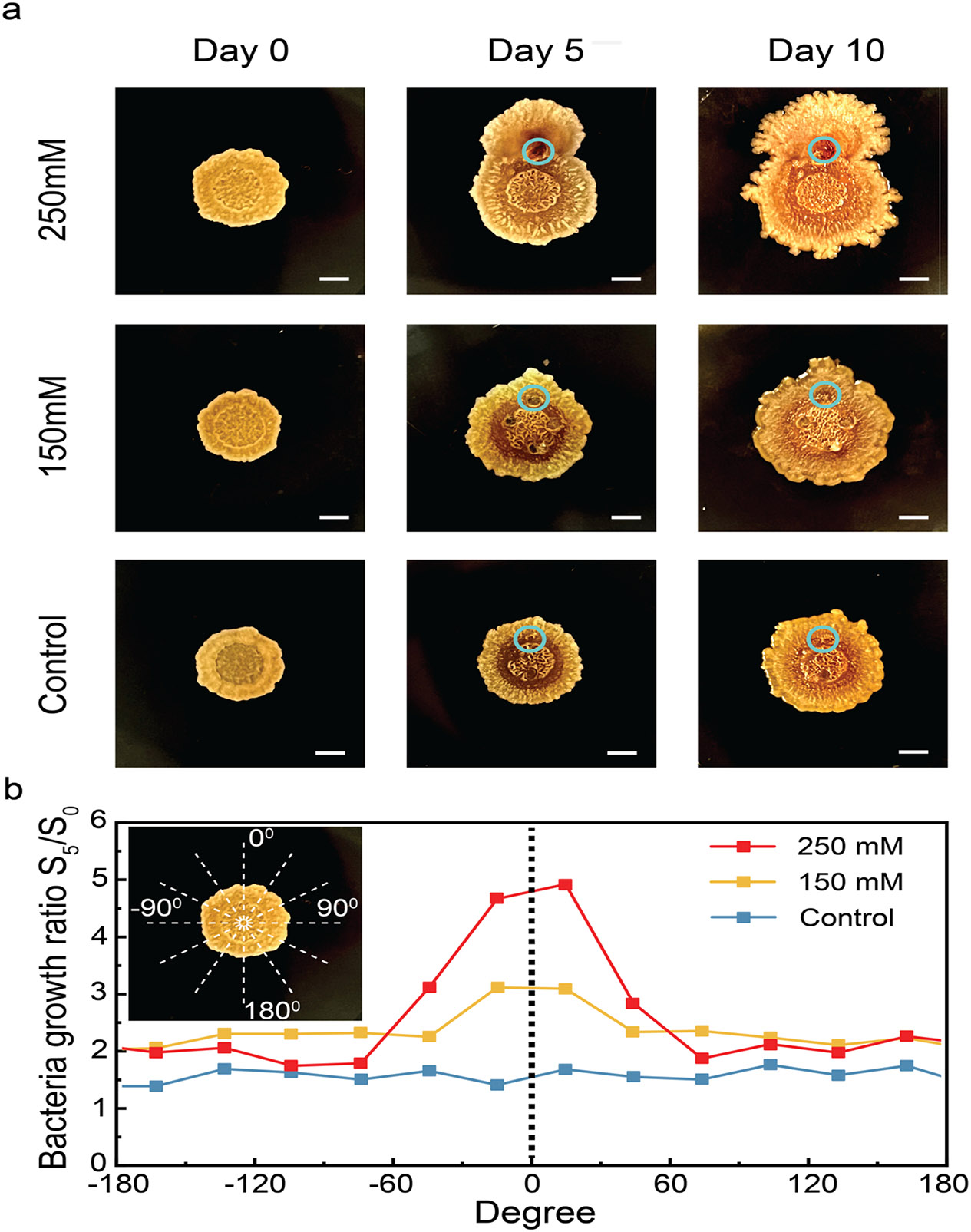
**a** Different dosage of K^+^ are delivered to the biofilm in spatial controlled manner. Blue circle denotes the location of WE. Sequential images capturing the biofilm at different time points, emphasizing the impact of K + delivery on directional growth. Scale bar = 2 mm. **b** Biofilm is segmented into 12 sections, with each section spanning 30° (inset). The size of the biofilm in each section is determined by the area it covers in the corresponding section. Bacterial growth ratio is depicted as the ratio between the size of the biofilm on day 5 and day 0. Here, we adopt the midpoint angle in each section to represent its corresponding bacterial growth ratio within the section. The black dashed line denotes the direction of the Working Electrode (WE)

The addition of K^+^ to the extracellular environment significantly impacts biofilm growth [18, 19]. Studies indicate that potassium ions play a crucial role in cell-to-cell signaling and metabolic coordination within the biofilm. We observed growth discrepancies between biofilms exposed to K^+^ and control (Fig. 4). K^+^ exposure at day 0 induces enhanced film growth observed at day 5 and day10 compared to control, and this enhanced growth is dose dependent (Fig. 4a). A Δ[K^+^] = 250 mM at day 0 induces a larger growth towards the WE (marked as a teal circle) compared to Δ[K^+^] = 150 mM both at day 5 and day 10 (Fig. 4a). To quantify this observation, we divide the image into 12 slices representing 30° each, with 0-degree representing the direction that connects the WE with the center of the film (see inset Fig. 4b).We then measure the number of pixels that correspond to the biofilm and normalize it to the figure acquired at day 0. As expected, growth in the control sample is isotropic. In this This striking result demonstrates the impact of delivering ions with spatial resolution. To quantify growth, we define the bacteria growth ratio as the ratio between the area covered by the biofilm on day 5 and the area covered on day 0. The areas close to the WE have an anisotropy ratio of 2.5 and 1.5 for Δ[K^+^] = 250 mM and 150 mM respectively. This result is remarkable and indicates the ability for the bioelectronic delivery of K^+^ to direct biofilm growth both in time and in space.

## Conclusion

We have designed a bioelectronic system that regulates membrane voltage and growth dyanamics in *B. subtilis* biofilms using fluxes of K^+^ delivered with an ion-pump. With determinist delivery of K^+^ we adjust the concentration of extracellular K^+^ and affect both the biofilm membrane voltage and growth dynamics. These effects are concentration dependent with higher K^+^ concentration and larger growth rate. We direct growth into desired directions and induce anisotropy in the biofilm. In the future, our platform can be used for the concurrent delivery of multiple ions and molecules, facilitating investigations into their collective impact on bacterial biofilm growth and the intricate mechanisms of cell-to-cell communication within these communities. These observations also offer valuable insights into using bioelectronics to shaping the electronic dynamics of bacterial cells and steering the morphology of bacterial colonies. In the future, similar strategies can be applied to mammalian cells to induce morphogenesis into desired shapes for potential applications in regenerative medicine.

## Methods

### Device Fabrication

The ion pump was fabricated using PDMS based soft lithography. We fabricated PDMS molds using Preform software and Form3 3D printers. These molds consist of two layers: the bottom layer that defines the reservoirs, and a top layer that serves as a lid to seal the reservoirs. The molds are filled with PDMS (mixed 10:1) and the PDMS is demolded after curing at 60°C for 48 h. Following demolding, we inserted Ag and AgCl wires, each with a diameter of 0.25 mm, into the reservoirs to create the electrodes. The Ag wires serve as the working electrodes, and the AgCl wires serve as the reference/counter electrodes.

Subsequently, we bonded the two layers of PDMS together, treating the contact interfaces with 50 W oxygen plasma for 30 s and securing them with custom-made aluminum clamps for 30 min. After bonding, we deposited a 1.5 μm thick water-insulating layer of Parylene-C using a Specialty Coating Systems Lab Coater, employing an A174 adhesion promoter to facilitate adhesion. This layer also prevents bubble formation within the reservoirs.

Post Parylene coating, we inserted four 5 mm long, 800 μm inner diameter glass capillaries filled with hydrogel through pre-made channels in the PDMS, and the reservoirs were filled with 1 M KCl solution using a syringe. An anionic hydrogel consisting of 2-Acrylamido-2-methylpropane sulfonic acid (AMPSA)—Poly (ethylene glycol) diacrylate (PEGDA) was used. This solution comprises the acrylate monomer mixed with PEGDA and a photo initiator (2-Hydroxy-4’-(2-hydroxyethoxy)-2-methylpropiophenone) to promote crosslinking in the presence of UV light. The procedures for preparing this solution have been previously documented [32]. All chemicals were purchased from Sigma-Aldrich.

A custom-made PCB was then soldered onto the PDMS device, with silver conductive paste and alloy dowel pins connecting the PCB to the electrodes before soldering to complete the connections. (Supplementary Fig. S1).

To anchor the completed device in six-well cell culture plates, we used a custom-made 3D printed adapter.

### Biofilm Formation

A non-domesticated biofilm-forming strain of *B. subtilis* (NCIB 3610) was used for all experiments. In brief, cells from a frozen stock were streaked on LB agar plates and incubated at 37 °C overnight. Then, a single colony was picked from the plate and grown in liquid LB to the mid-exponential phase. A drop of cells was placed on 1.5% agar pads made with MSgg medium [5 mM potassium phosphate (pH 7.0), 100 mM 3-(N-morpholino) propane sulfonic acid (pH 7.0), 2 mM MgCl2, 700 μM CaCl2, 50 μM MnCl2, 100 μM FeCl3, 1 μM ZnCl2, 2 μM thiamine, 0.5% glycerol, 0.5% glutamate] and 10 μM Thioflavin T (ThT) in a 6-well plate. The plate was maintained at room temperature for four days, and then incubated at 30 °C in a thermobox and cell growth was monitored using time-lapse fluorescence microscopy using an Olympus IX81 microscope and a 2.5 × objective.

### Fluorescence Probes

We used microscope-based real-time imaging to monitor the change in [K^+^] and V_mem_. The fluorescent probe ION Potassium Green-4 (IPG-4) TMA+ salt (3023F, ION biosciences, Texas) is a yellow-green, fluorescent, extracellular potassium ion indicator with λ_excitation_/λ_emission_ of 525 nm/545 nm respectively and has high sensitivity to detect small changes in K^+^ concentration. It exhibits a linear relationship between fluorescence intensity and K^+^ concentration (Supplementary Fig. S3). The dye was made to 3 mM dispensed in 0.1 M Tris buffer. All fluorescence images were analyzed using ImageJ software.

To observe the changes in cell membrane voltage (V_mem_), we used the fluorescent, cationic dye Thioflavin-T (ThT). ThT has an λ_excitation_/λ_emission_ of 349 nm/454 nm. All cell images were analyzed using ImageJ software.

### Time-Lapse Microscopy

Keyence BZ-X710 with a 2.5X objective was used for the experiments. Fluorescence images were taken with a YFP filter to characterize K^+^ delivery from the devices. Biofilm images were taken in phase contrast and CFP fluorescence. Images were taken every 10 min and the minimum exposure time to provide a good signal-to-noise ratio was used.

### COMSOL Simulations

K^+^ delivery was modelled by generating a diffusion model in COMSOL using Fick’s laws of diffusion. The diffusion coefficient of K^+^ in agar was considered as 1.6 × 10~^9^ m^2^ s^−1^. The initial [K^+^] was taken as 0, and we used the I_K+_ = 100 μA to generate the value for electric flux through an 800 μm diameter circular opening.

## Supplementary Material

Supplemental Doc

## Data Availability

The data that support the findings of this study are available from the corresponding author upon reasonable request.

## References

[1] Blackiston DJ, Mclaughlin KA, Levin M, Bioelectric controls of cell proliferation: Ion channels, membrane voltage and the cell cycle. Cell Cycle 8(21), 3527–3536 (2009) 19823012 10.4161/cc.8.21.9888 PMC2862582

[2] Levin M, Selberg J, Rolandi M, Endogenous bioelectrics in development, cancer, and regeneration: Drugs and bioelectronic devices as electroceuticals for regenerative medicine. Iscience 22, 519–533 (2019) 31837520 10.1016/j.isci.2019.11.023 PMC6920204

[3] Gerstner W, Kistler WM, Spiking Neuron Models: Single Neurons, Populations Plasticity (Cambridge University Press, UK, 2002)

[4] AbdulKadir L, Stacey M, Barrett-Jolley R, Emerging roles of the membrane potential: Action beyond the action potential. Front. Physiol 9, 1661 (2018). 10.3389/Fphys.2018.01661 30519193 PMC6258788

[5] Levin M, Pezzulo G, Finkelstein JM, Endogenous bioelectric signaling networks: Exploiting voltage gradients for control of growth and form. Annu. Rev. Biomed. Eng 19, 353–387 (2017). 10.1146/Annurev-Bioeng-071114-040647 28633567 PMC10478168

[6] Galera-Laporta L, Comerci CJ, Garcia-Ojalvo J, Süel GM, Ionobiology: The functional dynamics of the intracellular metallome, with lessons from bacteria. Cell Syst. 12(6), 497–508 (2021) 34139162 10.1016/j.cels.2021.04.011 PMC8570674

[7] Prindle A, Liu J, Asally M, Ly S, Garcia-Ojalvo J, Süel GM, Ion channels enable electrical communication in bacterial communities. Nature 527(7576), 59–63 (2015) 26503040 10.1038/nature15709 PMC4890463

[8] Kikuchi K , Electrochemical potential enables dormant spores to integrate environmental signals. Science 378(6615), 43–49 (2022) 36201591 10.1126/science.abl7484 PMC10593254

[9] Comerci CJ , Localized electrical stimulation triggers cell-type-specific proliferation in biofilms. Cell Syst. 13(6), 488–498 (2022) 35512710 10.1016/j.cels.2022.04.001 PMC9233089

[10] Kim J, Park HD, Chung S, “Microfluidic approaches to bacterial biofilm formation,” (In Eng). Molecules 17(8), 9818–9834 (2012). 10.3390/Molecules17089818 22895027 PMC6268732

[11] Yuan L, Straub H, Shishaeva L, Ren Q, “Microfluidics for biofilm studies,” (In Eng). Annu. Rev. Anal. Chem. (Palo Alto Calif) 16(1), 139–159 (2023). 10.1146/Annurev-Anchem-091522-103827 37314876

[12] Straub H, Eberl L, Zinn M, Rossi RM, Maniura-Weber K, Ren Q, A microfluidic platform for in situ investigation of biofilm formation and its treatment under controlled conditions. J. Nanobiotechnol 18(1), 166 (2020). 10.1186/S12951-020-00724-0 PMC7661213 33176791

[13] Berggren M, Richter-Dahlfors A, Organic bioelectronics. Adv. Mater 19(20), 3201–3213 (2007)

[14] Simon DT, Gabrielsson EO, Tybrandt K, Berggren M, Organic bioelectronics: Bridging the signaling gap between biology and technology. Chem. Rev 116(21), 13009–13041 (2016) 27367172 10.1021/acs.chemrev.6b00146

[15] Pitsalidis C , Organic bioelectronics for in vitro systems. Chem. Rev 122(4), 4700–4790 (2021) 34910876 10.1021/acs.chemrev.1c00539

[16] Jafari M, Marquez G, Dechiraju H, Gomez M, Rolandi M, Merging machine learning and bioelectronics for closed-loop control of biological systems and homeostasis. Cell Rep. Phys. Sci 4(8), 101535 (2023). 10.1016/J.Xcrp.2023.101535

[17] Williamson A , Controlling epileptiform activity with organic electronic ion pumps. Adv. Mater 27(20), 3138–3144 (2015) 25866154 10.1002/adma.201500482

[18] Arbring Sjöström T , Iontronics: A decade of iontronic delivery devices. Adv. Mater. Technol 3(5), 1870018 (2018)

[19] Strakosas X , Bioelectronic modulators: a bioelectronic platform modulates Ph in biologically relevant conditions (Adv. Sci. 7/2019). Adv. Sci 6(7), 1970041 (2019) 10.1002/advs.201800935 PMC6446605 30989015

[20] Selberg J , Machine learning-driven bioelectronics for closed-loop control of cells. Adv. Intell. Syst 2(12), 2000140 (2020)

[21] Poxson DJ , Capillary-fiber based electrophoretic delivery device. Acs Appl. Mater. Inter 11(15), 14200–14207 (2019). 10.1021/Acsami.8b22680 30916937

[22] Nguyen T, Asefifeyzabadi N, Li H, Luo L, Rolandi M, The Importance of electrode material in bioelectronic electrophoretic ion pumps. Adv Mater Technol 8(13), 2201996 (2023). 10.1002/Admt.202201996

[23] Dechiraju H , On-chip on-demand delivery of k+ for in vitro bioelectronics. Aip. Adv 12(12), 125205 (2022)

[24] Park Y , Modulation of neuronal activity in cortical organoids with bioelectronic delivery of ions and neurotransmitters. Cell Rep. Meth 4(1), 100686 (2024) 10.1016/j.crmeth.2023.100686 PMC10831944 38218190

[25] Marquez G , (2023). "Delivering biochemicals with precision using bioelectronic devices enhanced with feedback control," Biorxiv, P. 2023.08.29.555386, 10.1101/2023.08.29.555386. PMC11093312 38743674

[26] Jia M , Bioelectronic control of chloride ions and concentration with Ag/Agcl contacts. Apl. Mater 8(9), 091106 (2020)

[27] Jia M, Jafari M, Pansodtee P, Teodorescu M, Gomez M, Rolandi M, A multi-ion electrophoretic pump for simultaneous on-chip delivery of H+, Na+, And Cl−. Apl. Mater 10(4), 041112 (2022). 10.1063/5.0084570

[28] Yang C-Y , Encoding membrane-potential-based memory within a microbial community. Cell Syst. 10(5), 417–423 (2020) 32343961 10.1016/j.cels.2020.04.002 PMC7286314

[29] Liu J , Metabolic co-dependence gives rise to collective oscillations within biofilms. Nature 523(7562), 550–554 (2015) 26200335 10.1038/nature14660 PMC4862617

[30] Liu J , A sensitive and specific nanosensor for monitoring extracellular potassium levels in the brain. Nat. Nanotechnol 15(4), 321–330 (2020) 32042163 10.1038/s41565-020-0634-4

[31] Rana PS , Calibration and characterization of intracellular asante potassium green probes, Apg-2 And Apg-4. Anal. Biochem 567, 8–13 (2019) 30503709 10.1016/j.ab.2018.11.024

[32] Jia M, Luo L, Rolandi M, Correlating ionic conductivity and microstructure in polyelectrolyte hydrogels for bioelectronic devices. Macromol. Rap. Commun 43(6), 2100687 (2022). 10.1002/Marc.202100687 35020249

[33] Schantz EJ, Lauffer MA, Diffusion measurements in agar gel. Biochem. 1(4), 658–663 (1962) 14498029 10.1021/bi00910a019

[34] Whatmore AM, Chudek JA, Reed RH, The effects of osmotic upshock on the intracellular solute pools of bacillus subtilis. Microbiol. 136(12), 2527–2535 (1990). 10.1099/00221287-136-12-2527 2127802

[35] Milo R, Phillips R, Cell Biology By The Numbers (Garland Science, New York, 2015)

