# Supplementary material for: Bioelectronic Delivery of Potassium Ions Controls Membrane Voltage and Growth Dynamics in Bacteria Biofilms
Source: Biomed Mater Devices. Author manuscript; Available in PMC 2025 Jul 27. (PMC12291149; doi:10.1007/s44174-024-00209-w)
Supplement: Supplemental Doc [file NIHMS2023725-supplement-Supplemental_Doc.docx]

Biomedical Materials & Devices SUPPLEMENTARY INFORMATION

### **Bioelectronic delivery of potassium ions controls membrane voltage and growth dynamics of bacteria biofilms**

### Harika Dechiraju*, Yixiang Li*, Colin Comerci, Le Luo, Niloofar Asefi, Sydnie Figuerres, Ansel Trevino, Alexie Barbee, Maryam Tebyani, Prabhat Baniya, Mircea Teodorescu, Gurol Suel, Marco Rolandi

1. Fabrication Schematic


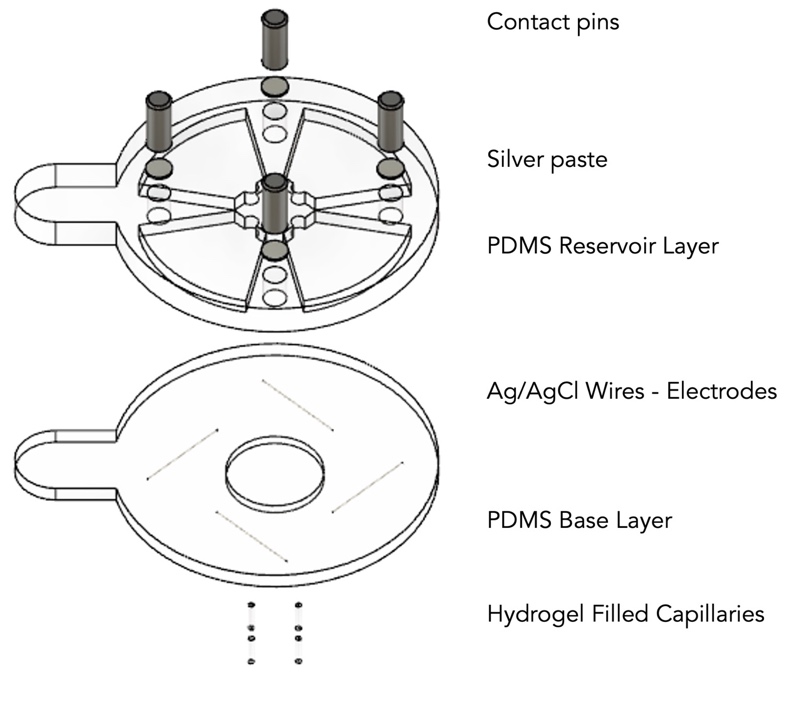


Figure S1 Fabrication schematic of the ion pump. Reproduced with permission from [36]

1. Optimization of K+ delivery

In order to achieve a higher delivery dosage, it is crucial to optimize the delivery parameters, such as the current produced and the duration of delivery. According to Ohm’s Law, the current produced is primarily dictated by the voltage, V_K+_, and the resistances in the circuit. Fig 2 shows a simplified circuit diagram of the ion pump schematic. The main resistances that contribute to the overall resistance of the ion pump include the resistance of the electrodes, electrolytes in the reservoir, and the ion exchange membrane. The effect of reservoir concentration and V_K+_ on delivery efficiency has previously been discussed in a study by Dechiraju et. al [23]. Here, we will discuss how to optimize the resistance of the electrodes and the ion exchange membrane to ensure that we can meet the required delivery dosage.

$$V=IR$$

$$R_{total}=R_{electrode}+ R_{electrolyte}+ R_{IEM}$$

Resistance depends on the resistivity (r), length (L), and cross-sectional area (A)

$$R= r\frac{L}{A}$$

For a given length and material of a conductor, increasing the cross-sectional area results in a reduction of the resistance.


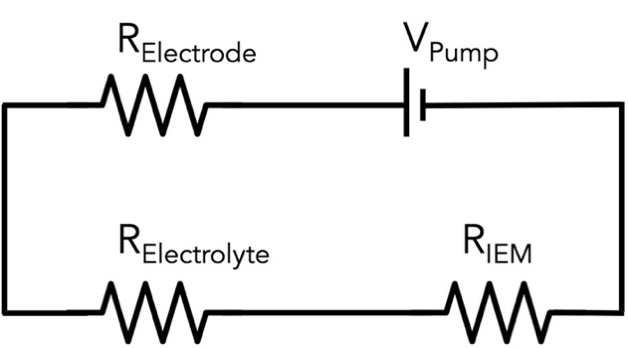


Figure S2 Circuit diagram of ion pump with the associated resistances

To reduce resistance associated with the electrodes (R_electrode_), we increased the thickness of the wire used from 0.1 mm diameter to 0.25 mm diameter. To reduce the resistance associated with the hydrogel (R_IEM_), we increased the thickness of the glass capillary from a 100 µm inner diameter to 800 µm inner diameter. The change in the current produced at each step is tracked and reported in table 1.

*Table 1: Comparison of current for different electrode and IEM diameters*

| Diameter of electrode (mm) | Diameter of capillary (µm) | Current, I (µA) |
| --- | --- | --- |
| 0.1 | 100 | 2 |
| 0.1 | 400 | 12 |
| 0.1 | 800 | 20 |
| 0.25 | 800 | 80 |

We observe 40 times increase in the current produced when the electrode diameter is increased to 0.25 mm and the capillary diameter is increased to 800 µm.

1. Calibration curve for IPG-4


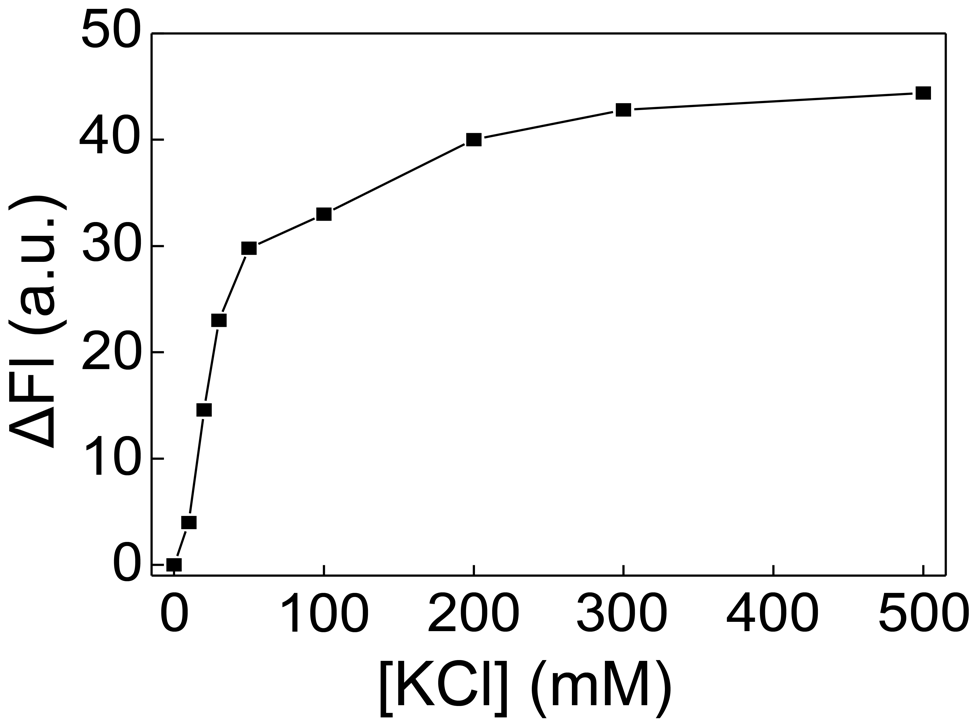


*Figure S3 Calibration curve of IPG-4 generated using KCl solution with known concentrations showing the relationship between [K+] and fluorescence intensity of IPG-4 dye*

1. Fluorescence response of the ion pump


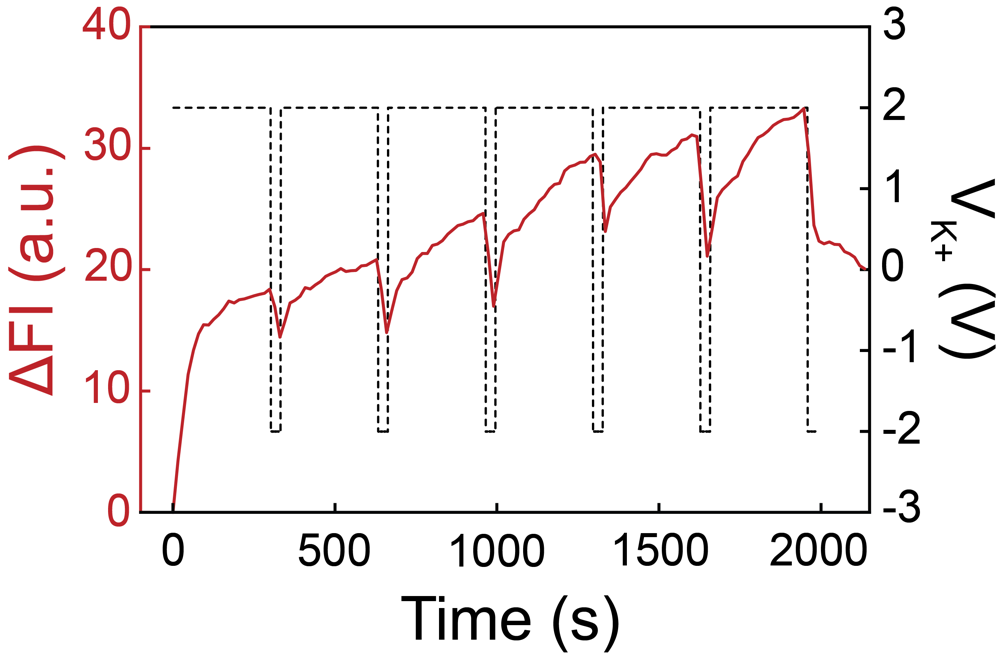


*Figure S4 Fluorescence response of the ion pump captured using IPG-4 dye for a series of alternating V_K+_ over 6 cycles. Based on the calibration curve, the total change of K^+^ concentration during the 6 cycles is approximately 100 mM.*

1. [K^+^] as a function of time

*
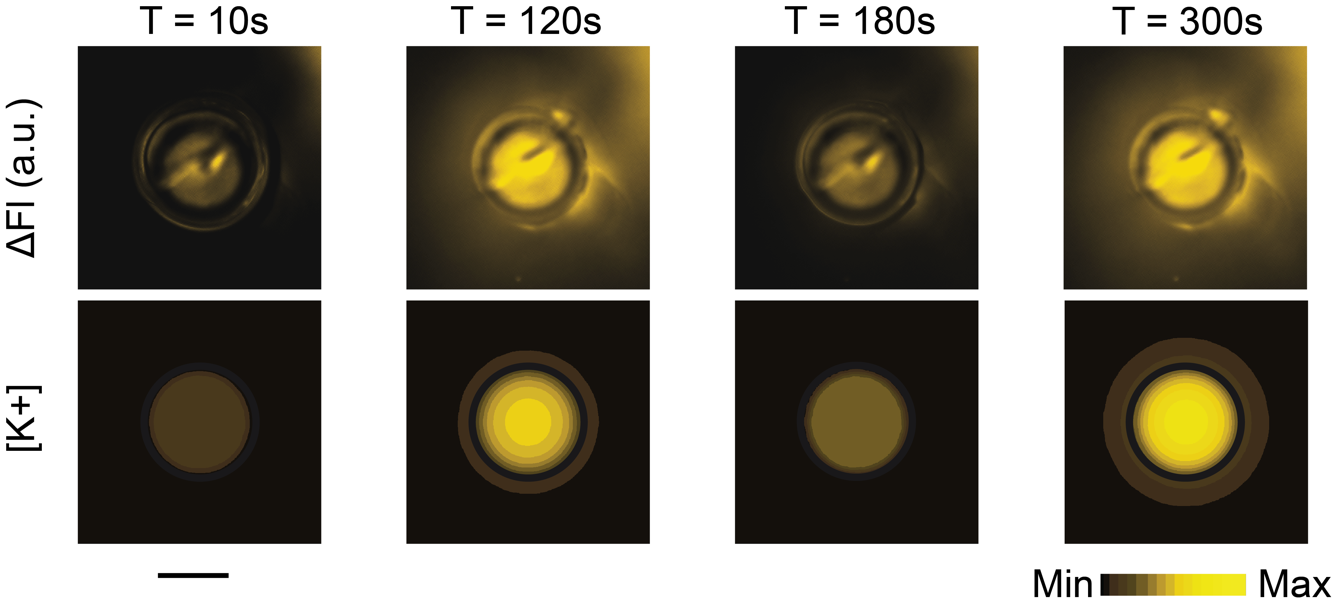
*

*Figure S5 Fluorescent microscope images capturing WE at various stages throughout the actuation cycle (top row), showing the On-Off trend due to the change of [K^+^]. Corresponding COMSOL simulation results depicting the change in [K^+^] at the WE (bottom row). Scale bar = 500 µm.*

1. Calibration curve of [K+]

*
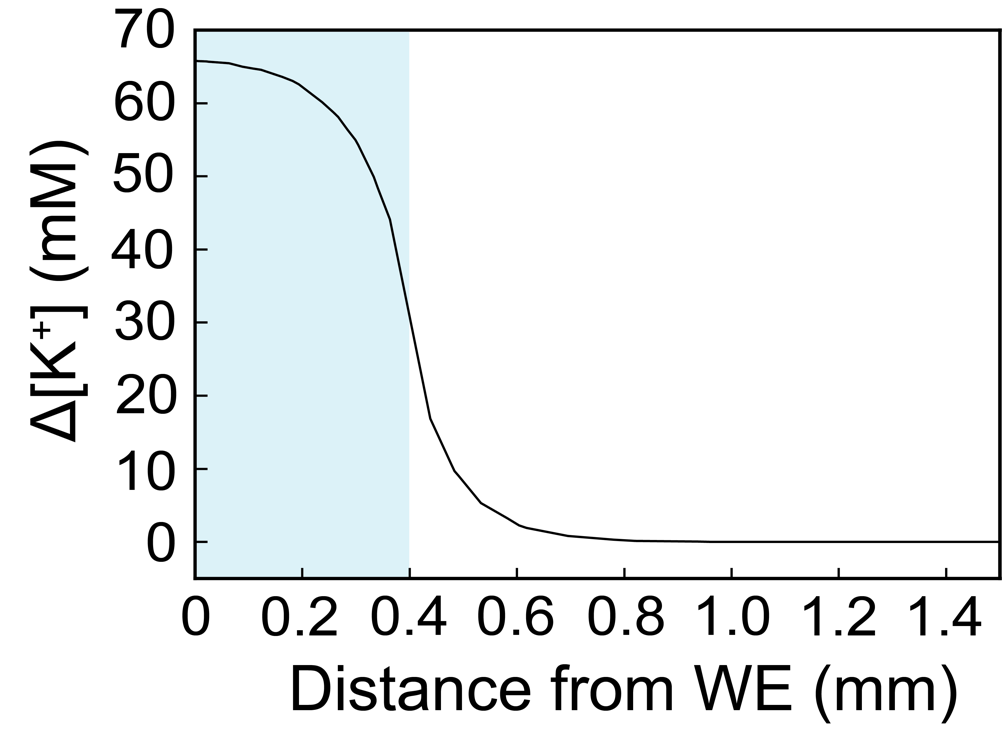
*

*Figure S6 COMSOL simulation depicting [K^+^] a function of the distance from the center of WE. The blue shading represents the area of WE.*

1. Biofilm images with different [K+] using IPG-4


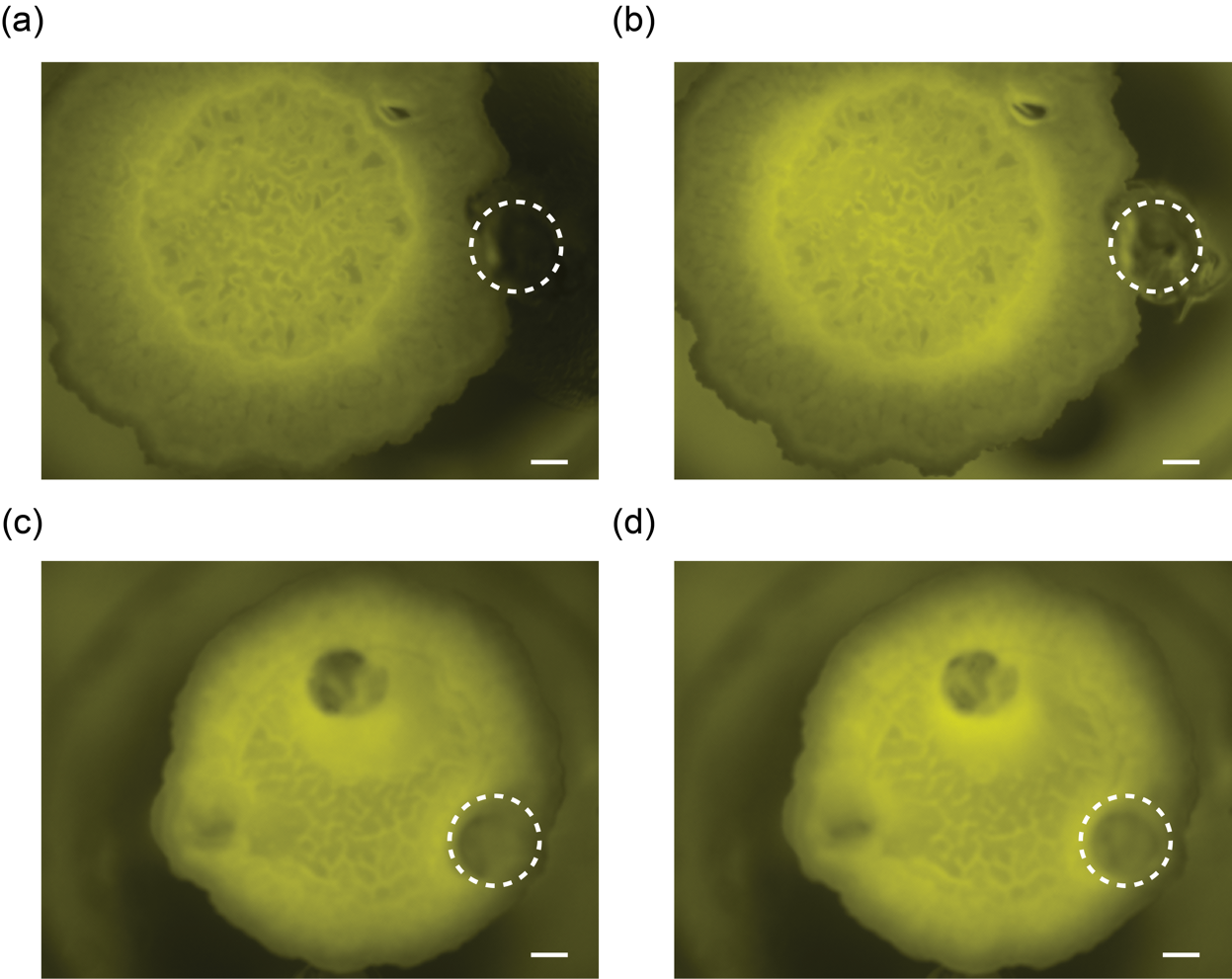


*Figure S7 Fluorescence image of the biofilm with IPG-4 dye. The top row are images under [K+] change of 250 mM. (a) the onset and (b) the conclusion. The bottom row are images under [K+] change of 150 mM. (c) the onset and (d) the conclusion. White dashed circle indicates the position of WE. Scale bar = 400 µm.*
